# Novel flat and wide meniscal repair material improves the ultimate load of knot breakage in a porcine trans-capsular meniscal repair model

**DOI:** 10.1186/s40634-017-0114-4

**Published:** 2017-12-19

**Authors:** Hiroyuki Yokoi, Tatsuo Mae, Ryo Iuchi, Yasuhiro Take, Yuta Tachibana, Kazunori Shimomura, Tomoki Ohori, Konsei Shino, Hideki Yoshikawa, Ken Nakata

**Affiliations:** 1 Medicine for Sports and Performing Arts, Department of Health and Sports Science, Osaka University Graduate School of Medicine, 2-2, Yamada-oka, Suita, Osaka, 565-0871 Japan; 2 Department of Orthopaedic Surgery, Osaka University Graduate School of Medicine, 2-2, Yamada-oka, Suita, Osaka, 565-0871 Japan; 3 Sports Orthopaedic Surgery Center, Yukioka Hospital, 2-2-3, Ukita, Kita-ku, Osaka, Osaka 530-0021 Japan

**Keywords:** Meniscal repair, Different structural sutures, Biomechanics, Knee

## Abstract

**Background:**

In the meniscal repair procedures, a high ultimate load capacity and low cyclic creep at the repair site are favorable and lead to good biological incorporation of the tear site after surgery. Previous biomechanical tensile tests of the meniscal sutures have identified the suture knot as the weakest point. We hypothesized that the strength of a suture knot depends on the suture shape, and therefore, we compared three differently shaped suture materials composed of the same material and quantity per length. The purpose of this study was to determine whether a novel flat and wide repair material (FWRM), which consists of braided multi-threads that are cross-sectionally flat and wide, improves the ultimate load of knot breakage in a biomechanical experiment using a porcine trans-capsular meniscal repair model.

**Methods:**

Eighteen fresh-frozen porcine knees (*n* = 6 in each group) were used. A longitudinal tear in the middle segment of the medial meniscus was created and repaired with a trans-capsular inside-out method using the following suture materials: No. 2–0 braided polyester conventional suture, hollow suture, and FWRM. After the separation of the inner segment of the meniscus with leaving, the suture stability of the repaired menisci was biomechanically analyzed with a video camera system for widening after a cyclic load between 5 and 20 N was applied 300 times. Ultimate failure load and stiffness at 5 mm/ min were also analyzed.

**Results:**

We found no significant difference in suture widening after cyclic load tests [conventional suture, mean 0.51 mm (S.D. 0.39 mm); hollow suture, mean 0.23 mm (S.D. 0.11 mm); and FWRM, mean 0.54 mm (S.D. 0.08 mm)]. The failure mode in all specimens was knot breakage. Compared with those of the other groups, the ultimate failure load of FWRM was statistically significantly higher in the load-to-failure tests (conventional suture, mean 58.8 N [S.D. 8.25 N]; hollow suture, mean 79.4 N [S.D. 10.2 N]; and FWRM, mean 97.4 N [S.D. 3.65 N]; *p* < 0.05).

**Conclusion:**

FWRM improves the ultimate load of knot breakage without altering stability. This material may contribute to safe and stable meniscus repair.

## Background

The meniscus in the knee joint plays key biomechanical roles in shock absorption, load transfer, lubrication, knee joint stabilization, and articular congruity increase (Barber and Stone, 1985; Hsieh and Walker, 1976; Kurosawa et al. 1980; Mac 1950; Voloshin and Wosk, 1983). Several studies have reported that osteoarthritis can develop in the knee joint after meniscal injury or meniscectomy (Baratz et al. 1986; Johnson et al. 1974; Krause et al. 1976). Arthroscopic meniscal repair has demonstrated good results with various suture techniques, such as the inside-out, outside-in, and all-inside techniques (Paxton et al. 2011; Tengrootenhuysen et al. 2011; Xu and Zhao, 2015). Although each meniscal repair technique has its advantages and disadvantages, the inside-out method remains the gold standard (Henning et al. 1988; Horibe et al. 1996; Horibe et al., 1995; Noyes and Barber-Westin 2002).

The effectiveness of a meniscal suture depends on adequate biomechanical stability at the repair site, the biological incorporation of the tear site, and the healing capacity of the meniscus (Post et al. 1997; Seil et al. 2000). In the meniscal repair procedures, stability at the repair site under a high ultimate tensile load and a low cyclic creep are favorable and leads to good biological incorporation of the tear site after surgery. The results of biomechanical tensile tests of meniscal sutures used with the inside-out technique have shown that the suture knot is the weakest point, and therefore, knot breakage occurs during ultimate failure load testing (Asik and Sener 2002; Barber et al. 2009; Barber and Stone, 1985; Brucker et al. 2010; Post et al. 1997; Seil et al. 2000; Zantop et al. 2005). These results prompted us to strengthen the ultimate load of the knot to achieve better meniscal repair results using the inside-out technique.

We hypothesized that altering of the cross-sectional shape of the sutures might improve the ultimate load of knot breakage. To test this hypothesis, we biomechanically analyzed three suture materials with different cross-sectional shapes composed of the same material and quantity per length using a previously described porcine trans-capsular meniscal repair model (Iuchi et al. 2017). These three suture materials included the conventional suture, which consists of a central core thread surrounded by thinly braided multi-threads; a novel hollow suture composed of braided multi threads without a central core thread; and a novel flat and wide repair material (FWRM) comprising braided multi-threads that are flat and wide in cross-section. The biomechanical characteristics of these suture materials under a cyclic load have not been described, and the ultimate tensile loads of these materials remain unknown. Therefore, the purpose of this study was to clarify the biomechanical characteristics of these suture materials when used with the inside-out technique in a porcine trans-capsular meniscal repair model.

## Methods

### Specimens and meniscus repair model

Eighteen porcine knees obtained from 6-month-old animals were used. All specimens were kept frozen at −20 °C and then allowed to thaw at 4 °C for 24 h. The medial meniscus was cut 20 mm longitudinally with a scalpel in the middle segment, leaving a peripheral meniscus 3 mm from the capsule (Kohn and Siebert 1989; Zantop et al. 2005) after the removal of the patella, patellar tendon, muscles, cruciate ligaments, lateral collateral ligament, lateral meniscus, and lateral half of the joint capsule. Trans-capsular inside-out meniscal repair was performed using three different suture materials as previously described (Iuchi et al. 2017).

**Fig. 1:**
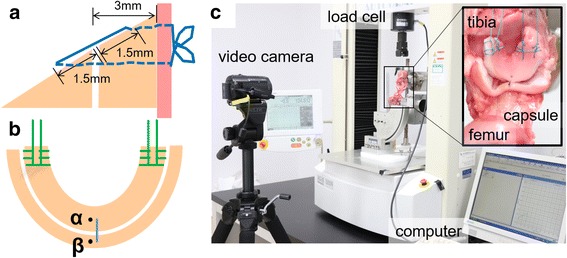
Inside-out meniscus repair procedure and biomechanical experiment methods. **a** A vertical suture initiated 3 mm from the inner edge of the meniscal lesion and meniscus was repaired within each 1.5-mm distance from the lesion. **b** Two points 1.5 mm away from the meniscal repair (points α and β) were marked and used to measure the distance after cyclic loading. **c** Biomechanical testing was conducted on a material testing machine and recorded with a video camera for analysis

The needles for meniscus repair were inserted at two points, each 1.5 mm from the meniscus tear site. Then, the suture materials were manually tied on the capsule with four square knots (Fig. 1a).

### Suture materials and experimental groups

**Fig. 2:**
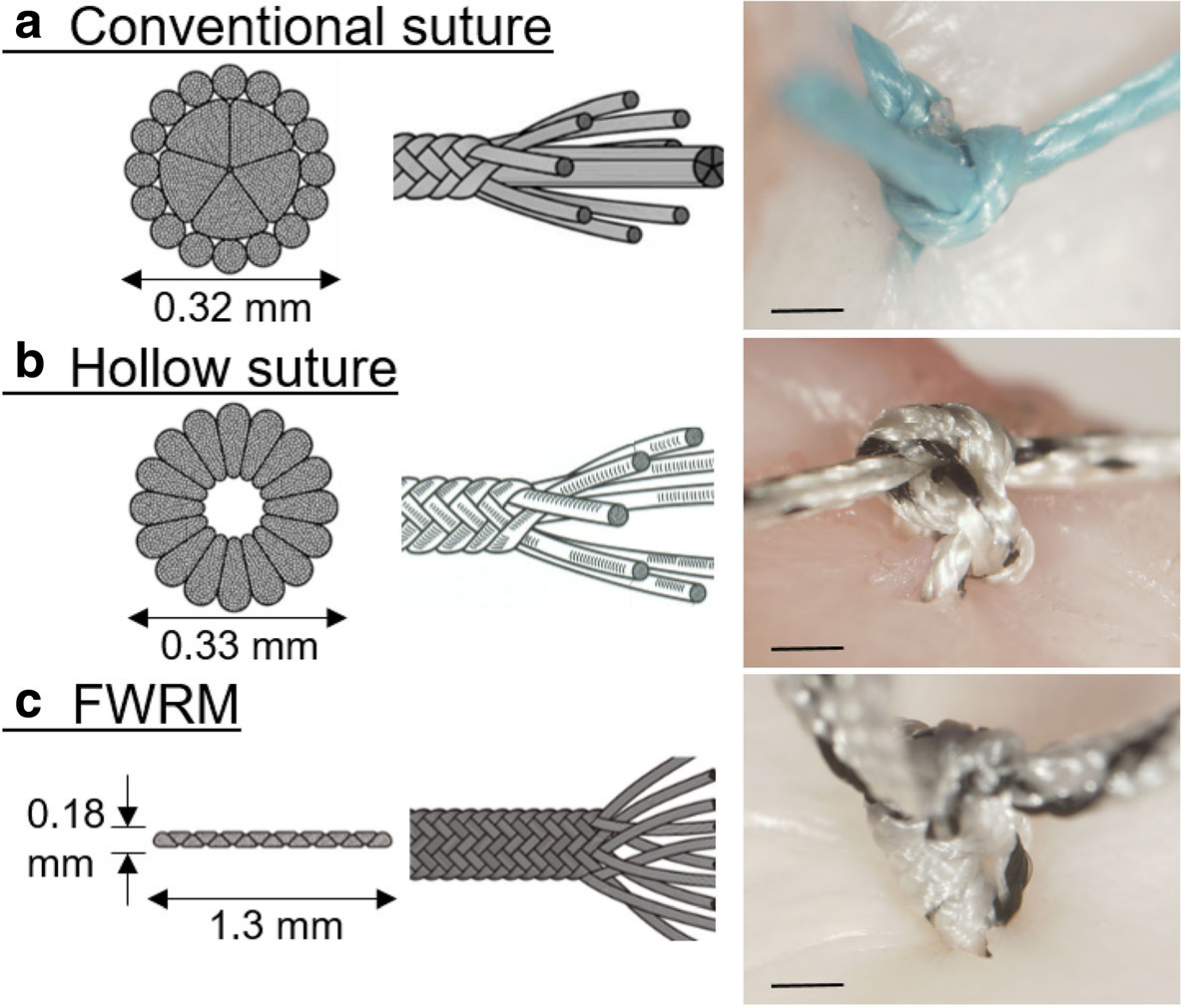
Structure of the three suture materials and their suture knots (bar: 0.5 mm). **a** Conventional suture: 2–0 braided polyester suture consisting of a core surrounded by threads with a total diameter of 0.32 mm. **b** Hollow suture: 2–0 braided polyester suture without a core thread and a total diameter of 0.33 mm. **c** Flat and wide repair material (FWRM): braided polyester suture with a flat form that had a height of 0.18 mm and width of 1.3 mm in cross-section

The eighteen specimens were divided into three groups according to the type of suture material used. Each type of suture consisted of the same quantity of polyester per length (Fig. 2).

The conventional suture group (C group, *n* = 6) used No. 2–0 braided polyester suture (Stryker Japan KK, Tokyo, Japan) consisting of a core thread surrounded by thin multi-threads with a total diameter of 0.32 mm (Fig. 2a). The hollow suture group (H group, *n* = 6) used No. 2–0 braided polyester suture (MENISCUS SUTURE 2–0 POLYESTER HOLLOW, Stryker Japan KK) composed of polyester multi-threads without a core thread. The diameter of each thread in the hollow suture was double that of the threads in the conventional suture. The hollow suture had a total diameter of 0.33 mm (Fig. 2b). The FWRM group (FW group, *n* = 6) used braided polyester material (MENISCUS SUTURE MESH, Stryker Japan KK) 0.18 mm high and 1.3 mm wise in cross-section (Fig. 2c). According to the manufacturer’s specifications (MATSUDAIKA KOGYO CO., LTD, Tokyo, Japan), the mean ultimate failure loads of the conventional suture, hollow suture, and FWRM are 41.7 N (S.D. 2.2 N), 67.0 N (S.D. 2.8 N), and 67.2 N (S.D. 4.2 N), respectively.

### Biomechanical tensile evaluation

Stability under cyclic loading, ultimate failure load, and stiffness during load-to-failure testing were biomechanically evaluated using a material testing apparatus, AUTOGRAPH AG-IS (SHIMADZU, Kyoto, Japan), as previously described (Iuchi et al. 2017). Briefly, the femur and tibia were set with custom holders, and No. 2 polyester sutures stitched at both ends of the meniscus were connected to the load cell through a clamp (Fig. 1b-c). A cyclic loading between 5 and 20 N was applied for 300 cycles, and a load-to-failure test was performed based on a minimum measurement of one-tenth of 1 mm, as previously described (Iuchi et al. 2017; Chang et al. 2005). To analyze cyclic creep, we marked two small dots adjacent to the suture insertion sites (Fig. 1b, α and β), and the motion of the two dots during cyclic loading was recorded with a video camera (HDR-CX370V; SONY, Tokyo, Japan). Then, the distance between the two dots was measured with image analysis software (DIPP-Motion Pro2D; DITECT, Tokyo, Japan). The widening of the sutures was calculated as the difference in the distances between the dots at the first cycle and the last cycle under a load of 5 N. This method allowed us to measure the actual widening at the repair site excluding the slippage of each clamp and the stress-relaxation of the soft tissue around the knee (Rosso et al. 2011, 2014).

After the cyclic loading test, the load-to-failure test at 5 mm/min was performed, and the stiffness during the load-to-failure test was also calculated in the linear region of the load-displacement curve. The failure mode was then documented. The three groups were compared in terms of widening after the cyclic load, ultimate failure load, stiffness, and failure mode.

### Statistical analysis

The data for each suture type were compared using one-way analysis of variance followed by the Tukey post hoc test to detect significant differences with statistical analysis software (PASW Statistics 18.0: SPSS Chicago, IL, USA). The statistical significance was set at *p* < 0.05.

## Results

The mean widening of the repair site after the cyclic load test was 0.51 mm (S.D. 0.39 mm) in the C group, 0.23 mm (S.D. 0.11 mm) in the H group, and 0.54 mm (S.D. 0.08 mm) in the FW group. There were no significant differences among these three groups (Table 1). The ultimate failure loads showed a mean value of 58.8 N (S.D. 8.25 N) in the C group, 79.4 N (S.D. 10.20 N) in the H group and 97.4 N (S.D. 3.65 N) in the FW group. With respect to the ultimate failure load, the H group was significantly higher than that of C group (*p* < 0.01), and FW group was significantly higher than those of C and H groups (C group vs. FW group, *p* < 0.01; H group vs. FW group, *p* = 0.02; Table 1). In the load-to-failure test, failure in all groups occurred owing to knot breakage, but no mid-substance suture breakage, suture loosening, or meniscal cut-out was observed (Table 2). The mean stiffness was 27.1 N/mm (S.D. 7.70 N/mm) in the C group, 45.0 N/mm (S.D. 7.74 N/mm) in the H group, and 43.7 N/mm (S.D. 7.93 N/mm) in the FW group. Compared with those of the other groups, the stiffness value of the C group was significantly lower (C group vs. H group, *p* < 0.01; C group vs. FW group, *p* = 0.01; Table 1).

**Table 1:** Widening after a cyclic loading, ultimate failure load and stiffness of the suture materials

	Widening after cyclic loading (mm)	Ultimate failure load (N)	Stiffness (N/mm)
C group (*n* = 6)	0.51 ± 0.39	58.8 ± 8.25^a,b^	27.1 ± 7.70^d,e^
H group (*n* = 6)	0.23 ± 0.11	79.4 ± 10.2^a,c^	45.0 ± 7.74^d^
FW group (*n* = 6)	0.54 ± 0.08	97.4 ± 3.65^b,c^	43.7 ± 7.93^e^

All values are given as means ± S.D. There were no statistically significant differences in widening among the groups after the cyclic load test. However, there were statistically significant differences among the groups in ultimate failure load. Compared with that of the other groups, the stiffness of the C group was significantly lower

^a^, ^b^, ^d^: *p* < 0.01; ^c^: *p* = 0.02; ^e^: *p* = 0.01

**Table 2:** Failure modes

	Knot breakage	Suture breakage	Suture loosening	Meniscal cut-out
C group	6/6	0/6	0/6	0/6
H group	6/6	0/6	0/6	0/6
FW group	6/6	0/6	0/6	0/6

Failure in all groups occurred owing to knot breakage, and no mid-substance suture breakage, suture loosening, or meniscal cut-out was observed

## Discussion

In meniscal repair, suture stability, and strength are key factors in meniscal healing, and a more stable and stronger suture is favorable (Post et al. 1997; Seil et al. 2000). We analyzed the stability and ultimate strength of three differently shaped suture materials in a porcine trans-capsular meniscal repair model.

There were no statistically significant differences in suture widening among these suture materials after cyclic load testing. Although there was concern that the knot made with FWRM would become unstable because of its flatness, the results of the cyclic load test showed that FWRM was as stable as both the conventional and the hollow sutures.

Interestingly, compared with that of the hollow suture, the ultimate failure load of the meniscal repair with FWRM was significantly higher, although the ultimate failure load of the hollow suture was almost the same as that of the FWRM. It is reasonable to assume that the repair weakens at the knot because knotting damages the thread via kinking or twisting, or both. Different suture material shapes have different knot configurations, and the shape might affect the degree of suture damage that occurs at the knot. Indeed, FWRM had a higher ultimate load with a smaller standard deviation, which suggests that it is consistently less damaged by knotting.

To the best of our knowledge, this study is the first to demonstrate that suture material with a flat and wide shape made of the same material and quantity per length as conventional polyester suture can improve the ultimate failure load of knot breakage. This improvement may occur because round suture material induces more folding or kinking compared with that of a flat and wide material.

Compared with that of the conventional suture, the stiffness values of the hollow suture and FWRM were significantly higher. This result might be attributable to the fact that only the core thread of the conventional suture elongates during the load-to-failure test, whereas in the hollow suture and FWRM, the entire thread conveys tensile stress. This difference in elongation may confer the greater stiffness observed in the hollow suture and FWRM.

The FWRM may have two potential advantages in addition to its high ultimate failure load and stability after cyclic loading. First, the lower profile of FWRM may make it safer for use in articular cartilage. Second, the flat shape of FWRM increases its area of contact with the injured meniscus, thereby reducing contact stress. One previous study showed that drawing strong sutures in the meniscus caused meniscal injury through repetitive stress (Seil et al. 2000), and in a porcine model, nearly 20% of the failure modes were meniscal cut-outs with No. 2–0 Force Fiber (Teleflex Medical, Research Triangle Park, NC) (Masoudi et al. 2015). In the present study, the ultimate failure load of the FWRM was 96.6 N, which was higher than those of the other two sutures, although the failure mode in all of the suture types was knot breakage with no meniscal cut-out. This finding may support the second potential advantage of the flat shape of FWRM. Similar features of flat-shaped materials were described in the field of spine and shoulder surgery (Boyer et al. 2015; Fujita et al. 2006; Hamasaki et al. 2010; Liu et al. 2017; Takahata et al. 2007), with greater footprint contact pressure being reported in an in vitro biomechanical study (Liu et al. 2017) and a lower re-tear rate reported in a clinical study (Boyer et al. 2015). Injured menisci are vulnerable to damage and cuts by sutures. By reducing these risks, the novel FWRM may have advantages over conventional suture material in the clinical setting. However, these advantages have not yet been verified, and further studies are necessary.

This study has limitations. First, a porcine meniscus was used instead of a human meniscus. Most available cadavers are from elderly patients who are likely to have degenerated menisci that introduce the risk that the biomechanical results would vary among specimens. Because several studies have shown that porcine menisci have properties comparable to those of human menisci (Barber and Stone, 1985, 2009; Fantasia et al. 2012; Joshi et al. 1995; Buckland DM et al. 2015; Masoudi et al. 2015), we selected undamaged porcine menisci to perform our study with high precision. Second, we did not investigate the consequences of suture shape in meniscus repair after surgery because the outcomes in this study were measured at time zero after surgery. Third, we conducted our biomechanical analysis only on the middle segment of the medial meniscus, and a recent study indicated that the location of sutures in the meniscus may affect their biomechanical features (Tiftikci and Serbest 2017). Finally, we did not investigate all-inside devices or sutures made of ultra-high-molecular-weight polyethylene (UHMWPE). Many suture devices using UHMWPE have been developed recently, and they show both high ultimate failure loads in biomechanical testing (Asik and Sener 2002; Barber et al. 2009, 2012; Brucker et al. 2010; Chang et al. 2005; Gunes et al. 2009; Iuchi et al. 2017; Joshi et al. 1995; Buckland DM et al. 2015; Zantop et al. 2005) and good clinical results (Moatshe et al. 2017; Tiftikci and Serbest 2016). These novel devices made of UHMWPE may further improve ultimate failure loads if a suture shape such as that of FWRM is used.

## Conclusions

The results of this biomechanical experimental study comparing three different shaped suture materials with the same material and quantity per length showed no significant differences in stability after cyclic load testing, but FWRM had a significantly higher ultimate failure load in meniscal repair according to the load-to-failure test. The FWRM was superior to conventional and hollow sutures in terms of the ultimate load of knot breakage and may improve the stability of meniscal sutures.

## Abbreviations

FWRM: Flat and wide repair material
UHMWPE: Ultra-high molecular weight polyethylene

## References

[CR1] Asik M, Sener N (2002). Failure strength of repair devices versus meniscus suturing techniques. Knee Surg Sports Traumatol Arthrosc.

[CR2] Baratz ME, Fu FH, Mengato R (1986). Meniscal tears: the effect of meniscectomy and of repair on intraarticular contact areas and stress in the human knee. A preliminary report. Am J Sports Med.

[CR3] Barber FA, Herbert MA, Bava ED, Drew OR (2012). Biomechanical testing of suture-based meniscal repair devices containing ultrahigh-molecular-weight polyethylene suture: update 2011. Arthroscopy.

[CR4] Barber FA, Herbert MA, Schroeder FA, Aziz-Jacobo J, Sutker MJ (2009). Biomechanical testing of new meniscal repair techniques containing ultra high-molecular weight polyethylene suture. Arthroscopy.

[CR5] Barber FA, Stone RG (1985). Meniscal repair. An arthroscopic technique. J Bone Joint Surg Br.

[CR6] Boyer P, Bouthors C, Delcourt T, Stewart O, Hamida F, Mylle G, Massin P (2015). Arthroscopic double-row cuff repair with suture-bridging: a structural and functional comparison of two techniques. Knee Surg Sports Traumatol Arthrosc.

[CR7] Brucker PU, Favre P, Puskas GJ, von Campe A, Meyer DC, Koch PP (2010). Tensile and shear loading stability of all-inside meniscal repairs: an in vitro biomechanical evaluation. Am J Sports Med.

[CR8] Chang HC, Nyland J, Caborn DN, Burden R (2005). Biomechanical evaluation of meniscal repair systems: a comparison of the Meniscal viper repair system, the vertical mattress FasT-fix device, and vertical mattress ethibond sutures. Am J Sports Med.

[CR9] Fantasia F, Potalivo G, Placella G, Fantasia L, Cerulli G (2012). Meniscal sutures: biomechanical study of mulberry and horizontal loop techniques. J Orthop Traumatol.

[CR10] Fujita MDM, Xu Z, Puttlitz CM (2006). A biomechanical analysis of sublaminar and subtransverse process fixation using metal wires and polyethylene cables. Spine (Phila Pa 1976).

[CR11] Gunes T, Bostan B, Erdem M, Asci M, Sen C, Kelestemur MH (2009). Biomechanical evaluation of arthroscopic all-inside meniscus repairs. Knee Surg Sports Traumatol Arthrosc.

[CR12] Hamasaki TTN, Kim J, Okada M, Ochi M, Hutton WC (2010). Pedicle screw augmentation with polyethylene tape: a biomechanical study in the osteoporotic thoracolumbar spine. J Spinal Disord Tech.

[CR13] Henning CE, Clark JR, Lynch MA, Stallbaumer R, Yearout KM, Vequist SW (1988). Arthroscopic meniscus repair with a posterior incision. Instr Course Lect.

[CR14] Horibe S, Shino K, Maeda A, Nakamura N, Matsumoto N, Ochi T (1996). Results of isolated meniscal repair evaluated by second-look arthroscopy. Arthroscopy.

[CR15] Horibe SSK, Nakata K, Maeda A, Nakamura N, Matsumoto N (1995). Second-look arthroscopy after meniscal repair. Review of 132 menisci repaired by an arthroscopic inside-out technique. J bone joint Surg Br.

[CR16] Hsieh HH, Walker PS (1976). Stabilizing mechanisms of the loaded and unloaded knee joint. J Bone Joint Surg Am.

[CR17] Iuchi R, Mae T, Shino K, Matsuo T, Yoshikawa H, Nakata K (2017). Biomechanical testing of transcapsular meniscal repair. J Exp Orthop.

[CR18] Johnson RJ, Kettelkamp DB, Clark W, Leaverton P (1974). Factors effecting late results after meniscectomy. J Bone Joint Surg Am.

[CR19] Joshi MD, Suh JK, Marui T, Woo SL (1995). Interspecies variation of compressive biomechanical properties of the meniscus. J Biomed Mater Res.

[CR20] Kohn D, Siebert W (1989). Meniscus suture techniques: a comparative biomechanical cadaver study. Arthroscopy.

[CR21] Krause WR, Pope MH, Johnson RJ, Wilder DG (1976). Mechanical changes in the knee after meniscectomy. J Bone Joint Surg Am.

[CR22] Kurosawa H, Fukubayashi T, Nakajima H (1980) Load-bearing mode of the knee joint: physical behavior of the knee joint with or without menisci. Clin Orthop Relat Res (149):283–290 https://www.ncbi.nlm.nih.gov/pubmed/?term=Kurosawa+H%2C+Fukubayashi+T%2C+Nakajima+H+ 7408313

[CR23] Liu RW, Lam PH, Shepherd HM, Murrell GAC (2017). Tape versus suture in arthroscopic rotator cuff repair: biomechanical analysis and assessment of failure rates at 6 months. Orthop J Sports Med.

[CR24] Buckland DM, Sadoghi P, Wimmer MD, Vavken P, Pagenstert GI, Valderrabano V, Rosso C (2015). Meta-analysis on biomechanical properties of meniscus repairs: are devices better than sutures?. Knee Surg Sports Traumatol Arthrosc.

[CR25] Mac CM (1950). The movements of bones and joints; the synovial fluid and its assistants. J Bone Joint Surg Br.

[CR26] Masoudi A, Beamer BS, Harlow ER, Manoukian OS, Walley KC, Hertz B, Haeussler C, Olson JJ, Zurakowski D, Nazarian A, Ramappa AJ, DeAngelis JP (2015). Biomechanical evaluation of an all-inside suture-based device for repairing longitudinal meniscal tears. Arthroscopy.

[CR27] Moatshe G, Cinque ME, Godin JA, Vap AR, Chahla J, LaPrade RF (2017) Comparable outcomes after bucket-handle Meniscal repair and vertical Meniscal repair can be achieved at a minimum 2 Years follow-up. Am J Sports Med. 10.1177/0363546517719244:363546517719244 10.1177/0363546517719244 28806092

[CR28] Noyes FR, Barber-Westin SD (2002). Arthroscopic repair of meniscal tears extending into the avascular zone in patients younger than twenty years of age. Am J Sports Med.

[CR29] Paxton ES, Stock MV, Brophy RH (2011). Meniscal repair versus partial meniscectomy: a systematic review comparing reoperation rates and clinical outcomes. Arthroscopy.

[CR30] Post WR, Akers SR, Kish V (1997). Load to failure of common meniscal repair techniques: effects of suture technique and suture material. Arthroscopy.

[CR31] Rosso C, Kovtun K, Dow W, McKenzie B, Nazarian A, DeAngelis JP, Ramappa AJ (2011). Comparison of all-inside meniscal repair devices with matched inside-out suture repair. Am J Sports Med.

[CR32] Rosso C, Muller S, Buckland DM, Schwenk T, Zimmermann S, de Wild M, Valderrabano V (2014). All-inside meniscal repair devices compared with their matched inside-out vertical mattress suture repair: introducing 10,000 and 100,000 loading cycles. Am J Sports Med.

[CR33] Seil R, Rupp S, Kohn DM (2000). Cyclic testing of meniscal sutures. Arthroscopy.

[CR34] Takahata MIM, Abumi K, Kotani Y, Sudo H, Ohshima S, Minami A (2007). Comparison of novel ultra-high molecular weight polyethylene tape versus conventional metal wire for sublaminar segmental fixation in the treatment of adolescent idiopathic scoliosis. J Spinal Disord Tech.

[CR35] Tengrootenhuysen M, Meermans G, Pittoors K, van Riet R, Victor J (2011). Long-term outcome after meniscal repair. Knee Surg Sports Traumatol Arthrosc.

[CR36] Tiftikci U, Serbest S (2016). Repair of isolated horizontal meniscal tears with all-inside suture materials using the overlock method: outcome study with a minimum 2-year follow-up. J Orthop Surg Res.

[CR37] Tiftikci U, Serbest S (2017). Does the location of placement of meniscal sutures have a clinical effect in the all-inside repair of meniscocapsular tears?. J Orthop Surg Res.

[CR38] Voloshin AS, Wosk J (1983). Shock absorption of meniscectomized and painful knees: a comparative in vivo study. J Biomed Eng.

[CR39] Xu C, Zhao J (2015). A meta-analysis comparing meniscal repair with meniscectomy in the treatment of meniscal tears: the more meniscus, the better outcome?. Knee Surg Sports Traumatol Arthrosc.

[CR40] Zantop T, Eggers AK, Musahl V, Weimann A, Petersen W (2005). Cyclic testing of flexible all-inside meniscus suture anchors: biomechanical analysis. Am J Sports Med.

